# The correlation between serum progesterone levels and syringoma

**DOI:** 10.3389/fmed.2026.1862404

**Published:** 2026-07-23

**Authors:** Ifen Ayu Malinda, Imam Budi Putra, Ariyati Yosi

**Affiliations:** Department of Dermatology and Venereology, Faculty of Medicine, Universitas Sumatera Utara, Medan, Indonesia

**Keywords:** benign tumor, eccrine gland, hormonal correlation, progesterone, syringoma

## Abstract

**Background:**

Syringoma is a benign adnexal tumor that originates from the eccrine glands. Clinically, it manifests as multiple papules that range in size from 1 to 5 mm. These papules are typically skin-colored to yellowish and often appear in the periorbital region. Although not malignant, syringoma can be aesthetically disturbing and may affect an individual’s self-confidence. The etiopathogenesis of syringoma is not fully understood, but the influence of progesterone has been suspected as a possible cause.

**Objective:**

This study aims to determine the correlation between serum progesterone levels and syringoma.

**Methods:**

This is an observational cross-sectional study involving 35 patients with syringoma and 35 patients without syringoma. Each patient underwent anamnesis, dermatologic and dermoscopic examinations, and blood sampling to assess serum progesterone levels using the enzyme-linked immunosorbent assay (ELISA) test. The data were analyzed using Fisher’s exact test.

**Results:**

The majority of syringoma patients were found in the age range of 26–35 years, comprising 17 subjects (48.57%). The distribution of syringoma types showed that the most common was the localized form, found in 18 subjects (51.43%), followed by the familial form in 9 subjects (25.71%) and the generalized form in 8 subjects (22.86%). Analysis of serum progesterone levels indicated that among all study samples, 10 subjects (28.57%) with high progesterone levels belonged to the group syringoma, while only 2 subjects (5.71%) belonged to the control group. The results of this study demonstrate a positive correlation [OR 6.6 (95% CI 1.33–32.8), *p* = 0.023].

**Conclusion:**

Syringoma predominantly affects young to mid-adult women, with localized forms most common and familial cases present. The lesions frequently occur in the periorbital region, particularly on the lower eyelid. A strong association between elevated progesterone levels and progesterone receptor expression suggests hormonal involvement. Progesterone may influence tumor development through its effects on sweat glands, keratinocytes, and skin structure.

## Introduction

Syringoma derives from the Greek word “syrinx,” which means tube or channel. In the past, syringoma was believed to be a glandular organ, but with advancements in technology and the discovery of immunohistochemical examination and electron microscopy, it was discovered that syringoma is a benign tumor originating from the eccrine glands. The primary function of the eccrine glands is to regulate body temperature in humans (1, 2).

The prevalence of syringoma was found to be 0.6–1% of the population worldwide, with higher rates observed among Asians and Africans (3). Syringoma can occur in individuals of any age group, but is more common after puberty and can continue into adulthood, with most cases found in women. In a retrospective study conducted by Lim et al., it was reported that out of 94 syringoma patients in South Korea, there were more women (69.2%) than men (30.8%) (4).

The primary cause of syringoma is still unknown; however, there are several factors that contribute to its development, including hormonal influences, genetic predisposition, infections, sun exposure, and autoimmune issues. These factors lead to the proliferation of the spiral structures of the sweat glands, resulting in a blockage of the eccrine sweat glands. This blockage prevents sweat from reaching the surface of the skin (1, 5).

Clinical manifestations of syringoma can present as small, multiple, skin-colored or slightly yellowish papules that are symmetrically distributed and usually asymptomatic, although some patients report itching that precedes the onset of the lesions. The most common area affected by syringoma is the lower periorbital region; however, it can be found on the scalp, chest, penis, vulva, and axillary region (5–7). One way to diagnose syringoma is by dermoscopic examination (8). Dermoscopy of syringoma typically shows multiple small, round-to-oval, whitish-yellow homogeneous areas, sometimes featuring central keratin-filled pores and fine telangiectasias. These characteristics can help differentiate syringoma from other papular lesions (8).

Hormones such as progesterone, which are essential for skin health, may be linked to the formation of syringomas (9). Progesterone is one of the seven hormones identified in the skin, according to a study by Pineider et al. (10). The gonads, made up of the testes and ovaries, and the adrenal cortex typically produce progesterone, an endogenous steroid hormone. During the luteal phase, there is an increase in progesterone levels (11). By interacting with progesterone receptors, this hormone exhibits biological effects on the skin.

Several studies have explored the relationship between progesterone and syringoma. For example, an immunohistochemistry study by Fathy et al. found that progesterone receptor (PR) expression was present in the cell nuclei and cytoplasm of cells in 23 syringoma patients. This suggests that there is a cell-specific role of the PR that can modulate the expression of eccrine cells (9). Research by Wallace and Smoller, using immunohistochemistry, revealed that PR expression was found in 89% of syringoma patients (12). A case report by Timpanidis, Lakhani, and Groves also revealed PR expression in patients with syringoma, supporting the strong hypothesis that hormonal control may influence the pathogenesis of eruptive syringoma (13). The presence of PR expression in syringoma patients strongly indicates that circulating progesterone affects eccrine cells.

Syringoma is a benign tumor that is not life-threatening, but it can be aesthetically disturbing and affect one’s self-confidence. Defects in the facial skin can influence how people perceive themselves (14). According to Frankel, syringoma patients often feel worried because it affects their facial beauty (15).

The etiopathogenesis of syringoma remains unclear, so the management is based only on clinical symptoms and aims to be cosmetic rather than addressing the underlying on cause (15). The results of currently available syringoma management options are unsatisfactory and may lead to recurrence (16). One suspected cause of syringoma is the influence of the hormone progesterone; however, research showing the relationship between serum progesterone levels and syringoma has not yet been conducted. Therefore, researchers are interested in investigating this relationship.

## Methods

### Study design

This observational study employed a cross-sectional design and involved 70 female subjects who underwent an examination at the Padang Bulan Public Health Center in Medan from October 2023 to August 2024. Each subject underwent history taking, physical examination, and dermoscopic examination. Only female participants were enrolled to reduce hormonal heterogeneity, as progesterone-related pathophysiology and progesterone receptor expression have been more frequently reported and studied in women.

### Sample size

To calculate the sample size in this study, the following formula was used:


$$n_{1}=n_{2}=\frac{{\left[Z_{\alpha }\sqrt{2\mathrm{PQ}}+Z_{\beta }\sqrt{\left(P_{1}Q_{1})+(P_{2}Q_{2}\right)}\;\right]}^{2}\;}{{\left(P_{1}-P_{2}\;\right)}^{2}}$$

*n*_1_ = minimum sample size.

*Z*_α_ = standard value 𝛼 5% = 1.96.

*Z_β_* = standard value β 20% = 0.84.

*P*_1_ = proportion of syringoma based on literature = 0.88.

*Q_1_ = 1 - P_1_ = 1–0.88 = 0.12.*

*P_1_ – P_2_ =* The minimum proportion difference considered significant is set at.

*P_2_ = P_1_–0.3 = 0.88–0.35 = 0.53.*

*Q_2_ = 1 - P_2_ = 1–0.53 = 0.47.*

*P = (P_1_ + P_2_) / 2 = 0.705.*

*Q = 1 - p = 0.295.*

*n_1_ = n_2_ = 28.0905302897056 = 28.*

Based on the formula above, the sample size for this study would typically consist of 28 subjects in the syringoma group and 28 in the control group. However, in this study, the sample size has been increased to 35 subjects with syringoma and 35 controls.

### Inclusion and exclusion criteria

The inclusion criteria were female subjects aged ≥ 18 years old who had been diagnosed with syringoma based on history taking, clinical examination, and dermoscopic examination. Subjects have a normal menstrual cycle (menstrual cycle not below 21 days and not above 38 days) and are willing to be included in the study by signing informed consent. The exclusion criteria included subjects who are pregnant and breastfeeding, subjects who are using hormonal contraceptives and prescribed drugs in the last 3 months, and who are already in the Menopause phase.

### Data collection

History taking, physical and dermatological examination, and data recording of variables (such as the subjects’ age, gender, and underlying disorder) were conducted by researchers and the clinical diagnosis was made by an experienced dermatologist in Prof. Dr. Chairuddin P Lubis, University of Sumatera Utara Hospital. Dermoscopy was used to increase diagnostic confidence but routine histopathological confirmation was not performed because biopsies were considered cosmetically unacceptable for typical periorbital lesions; histopathology was reserved for clinically atypical or uncertain cases. Blood sampling was performed during the luteal phase, estimated to begin on the 14th day after the first day of the last menstrual period (LMP) and continue until the next menstrual period. This period corresponds to the post-ovulation phase, during which progesterone secretion from the corpus luteum peaks, ensuring physiologically high and relatively stable hormone levels in all subjects. The study subjects had 3 cc of blood samples collected. Blood samples were centrifuged at 2,000–3,000 revolutions per minute for 20 min to obtain serum. Progesterone levels were analyzed using a PROG ELISA KIT, Cat No E1007Hu. For interpretation in the luteal phase, values <2 ng/mL were considered low and values ≥ 22 ng/mL were considered high (lab reference/kits insert) (17).

### Ethical considerations

This study was conducted after obtaining ethical clearance from the Research Ethics Committee of the University of Sumatera Utara (No. 169/KEPK/USU/2023) and from the Prof. Chairuddin P Lubis Universitas Sumatera Utara Hospital Research Permit (No. 670/UN5.2.1.1.2.2.11/PPM/2023). All of the procedures were conducted in accordance with the Helsinki Declaration of 1975, as revised in 2013.

### Statistical analysis

All data collected and analyzed using IBM SPSS statistical software. Data processing was carried out using descriptive analysis to determine the frequency distribution of the research variables. Then proceed with inferential analysis to analyze the relationship between independent and dependent variables, in this case, to determine the relationship between serum progesterone levels and *syringoma* with Fisher’s exact test. Continuous variables are presented as mean ± SD or median (IQR) based on normality. Categorical variables are counts and percentages. Comparisons between groups were performed using Fisher’s exact test for categorical variables and the Mann–Whitney *U* test for continuous variables that were not normally distributed. The effect size for the association between high progesterone and syringoma is reported as an odds ratio (OR) with 95% confidence interval. Multivariable logistic regression was planned but not performed due to limited sample size and few events. A two-tailed *p*-value of < 0.05 was considered statistically significant.

## Results

The results showed that the mean age distribution of the syringoma group subjects was 34.22 years, with the youngest being 20 years old and the oldest being 48 years old, as shown in Table 1. Most syringoma patients were found in the age range of 26–35 years, comprising 17 subjects (48.57%). The demographic characteristics are shown in Tables 1, 2.

**Table 1:** Distribution of syringoma subjects by age.

Age (years old)	Syringoma groups, *n* (%)	**Mean ± SD**
18–25	5 (14.29)	34.2 ± 8.04 years
26–35	17 (48.57)	
36–45	10 (28.57)	
46–55	3 (8.57)	

**Table 2:** Characteristics of syringoma subjects based on family history.

Family history	*n*
Mother	8
Father	1
Sibling	3
None	26

The distribution of syringoma type showed that the most common was the localized form, found in 18 subjects (51.43%), followed by the familial form in 9 subjects (25.71%) and the generalized form in 8 subjects (22.86%), as shown in Table 3.

**Table 3:** Distribution of syringoma subjects by type.

Syringoma type	Syringoma groups, *n* (%)
Localized form	18 (51.43)
Familial form	9 (25.71)
Generalized form	8 (22.86)
A form associated with *Down’s syndrome*	–

Syringoma was most prevalent in the periorbital (inferior palpebral) region with 33 subjects, followed by the periorbital (orbital) region with 22 subjects and the infraorbital region with 10 subjects, as shown in Table 4.

**Table 4:** Characteristics of syringoma subjects based on syringoma location.

Syringoma type	*n*
Periorbital (orbital)	22
Periorbital (palpebral superior)	9
Periorbital (palpebral inferior)	33
Infraorbital	10
Nasal	2
Zygomaticus	2

Analysis of serum progesterone levels indicated that among all study samples, 10 subjects (28.57%) with high progesterone levels belonged to the group syringoma, while only 2 subjects (5.71%) belonged to the control group. The analysis revealed a significant correlation between serum progesterone levels and syringoma, with an OR (95% CI) of 6.6 (95% CI 1.33–32.8) and a *p*-value of 0.023. The correlation between serum progesterone levels and syringoma is shown in Table 5.

**Table 5:** Correlation between serum progesterone levels and syringoma.

Progesterone serum (ng/mL)	Syringoma groups, *n* (%)	Control groups, *n* (%)	OR (95% CI)	*p*-value
Normal	25 (71.43)	33 (94.29)	6.6 (95% CI 1.33–32.8)	0.023
High	10 (28.57)	2 (5.71)		

## Discussion

Syringoma is a benign tumor originating from the eccrine sweat glands, predominantly occurring in women and assumed to be affected by hormonal changes (1, 4). This study found that the highest incidence of syringoma is found in subjects between 26 and 35 years old (48.57%). The results of this study are in line with research conducted by Lei et al., which showed that 70% of syringoma patients were aged 20–40 years (18). The same condition was also found in research conducted by Ghanadan and Khosravi, who showed that out of 34 cases of syringoma, the age of most patients was in their third decade (5).

The most common clinical form observed in this study was localized syringoma (51.43%), followed by a familial presentation (25.71%). These findings reinforce the clinical heterogeneity of syringoma and the contribution of both sporadic and familial factors to its presentation. This is in line with research by Yahya, which states that localized syringoma is the most common type of syringoma, predominantly occurs in women, and appears in the lower eyelid or periorbital area (19).

Syringoma can involve only one region or multiple regions. Syringoma was most prevalent in the periorbital (inferior palpebral) region, with 33 subjects, followed by the periorbital (orbital) region, with 22 subjects, and the infraorbital region, with 10 subjects. This finding is in line with the results of research conducted by Bagatin, Enokiahara, and De Souza, who conducted research on 38 syringoma subjects, found that 27 subjects (71.1%) had syringoma lesions in the area around the under eye (20). Research conducted by Ghanadan and Khosravi also found that 55.8% of patients had syringoma lesions located in the periorbital area (5).

The correlation between syringoma and progesterone levels suggests that these hormone levels may play a role in the development of syringoma through mechanisms that affect sweat gland activity and structure. These findings are consistent with a study by Wallace and Smoller, who found progesterone receptor expression in 89% of syringoma patients (12). The study conducted by Fathi et al. on 25 syringoma patients also showed that there was PR (progesterone receptor) expression in 92% of the patients. The results of this study support the view that high expression of PR in syringoma cells indicates that these cells may act as targets for progesterone action (9).

Progesterone has various effects on the skin, including influencing sebum production and modulating immune responses. It can also affect the proliferation of keratinocytes. However, in some circumstances, particularly with hormonal imbalances or fluctuations, progesterone may contribute to abnormal skin conditions involving excess keratin production or irregular growth patterns in the skin (21). This exposure may make the skin more susceptible to changes in keratinocyte proliferation, possibly leading to conditions characterized by hyperkeratosis or abnormal skin thickening (21, 22). In this study, the highest progesterone level among the group of syringoma patients was 37.9 ng/mL. There were 10 subjects (28.57%) with high progesterone levels, who belonged to the group syringoma, while only 2 subjects (5.71%) belonged to the control group.

### Limitation

This was a single-center, cross-sectional study with a relatively small sample size, which limits causal inference and may have reduced statistical power. Progesterone levels vary across the menstrual cycle; although we restricted to women with regular cycles, blood sampling was not consistently timed to a specific cycle day. Diagnosis was based on clinical and dermoscopic criteria without routine histopathological confirmation. Potential confounders (age, menstrual phase, family history, BMI, other hormonal factors) were not adjusted for in multivariable models. These limitations should be considered when interpreting the findings.

### Future suggestion

Larger prospective studies with standardized sampling timing, histopathologic confirmation, multivariable adjustment for confounders, and exploration of progesterone receptor expression (IHC) are needed to confirm causality and elucidate mechanisms. If hormonal involvement is confirmed, therapeutic implications (e.g., hormonal modulation) could be explored.

## Conclusion

In this cross-sectional study, syringoma occurred most commonly in women aged 26–35 and predominantly presented as localized periorbital lesions. Elevated serum progesterone was more frequent among syringoma patients than controls (28.6% vs. 5.7%; OR 6.6, 95% CI 1.33–32.8; *p* = 0.023), suggesting a possible hormonal association. However, the cross-sectional design, small sample size, lack of routine histopathological confirmation, and absence of multivariable adjustment limit causal inference; larger prospective studies with timed sampling and PR expression assessment are needed.

## Data Availability

The raw data supporting the conclusions of this article will be made available by the authors, without undue reservation.
